# Extended FTLD pedigree segregating a Belgian *GRN*-null mutation: neuropathological heterogeneity in one family

**DOI:** 10.1186/s13195-017-0334-y

**Published:** 2018-01-22

**Authors:** Anne Sieben, Sara Van Mossevelde, Eline Wauters, Sebastiaan Engelborghs, Julie van der Zee, Tim Van Langenhove, Patrick Santens, Marleen Praet, Paul Boon, Marijke Miatton, Sofie Van Hoecke, Mathieu Vandenbulcke, Rik Vandenberghe, Patrick Cras, Marc Cruts, Peter Paul De Deyn, Christine Van Broeckhoven, Jean-Jacques Martin

**Affiliations:** 1 Institute Born-Bunge, Neuropathology and Laboratory of Neurochemistry and Behavior, University of Antwerp, Universiteitsplein 1, B-2160 Antwerp, Belgium; 2 Department of Neurology, Ghent University Hospital, Ghent, Belgium; 3 Neurodegenerative Brain Diseases Group, Center for Molecular Neurology, VIB , Universiteitsplein 1, B-2610 Antwerp, Belgium; 4 Laboratory of Neurogenetics, Institute Born-Bunge, University of Antwerp, Antwerp, Belgium; 5 Department of Neurology and Memory Clinic, Hospital Netwerk Antwerp (ZNA) Middelheim and Hoge Beuken, Antwerp, Belgium; 6 Department of Neurology, Antwerp University Hospital, Edegem, Belgium; 7 Department of Pathology, Ghent University Hospital, Ghent, Belgium; 8 Department of Electronics and Information Systems, Ghent University, Ghent, Belgium; 9 Department of Neurosciences, Faculty of Medicine, KU Leuven, Leuven, Belgium; 10 Department of Old Age Psychiatry and Memory Clinic, University Hospitals Leuven, Leuven, Belgium; 11 Department of Neurology, University Hospitals Leuven, Leuven, Belgium; 12 Department of Neurology and Alzheimer Research Center, University of Groningen and University Medical Center Groningen, Groningen, The Netherlands

**Keywords:** Frontotemporal lobar degeneration, FTLD, FTD-*GRN*, FTLD-TDP, Frontotemporal dementia, FTD, Cerebral small vessel disease (SVD)

## Abstract

**Background:**

In this paper, we describe the clinical and neuropathological findings of nine members of the Belgian progranulin gene (*GRN*) founder family. In this family, the loss-of-function mutation IVS1 + 5G > C was identified in 2006. In 2007, a clinical description of the mutation carriers was published that revealed the clinical heterogeneity among IVS1 + 5G > C carriers. We report our comparison of our data with the published clinical and neuropathological characteristics of other *GRN* mutations as well as other frontotemporal lobar degeneration (FTLD) syndromes, and we present a review of the literature.

**Methods:**

For each case, standardized sampling and staining were performed to identify proteinopathies, cerebrovascular disease, and hippocampal sclerosis.

**Results:**

The neuropathological substrate in the studied family was compatible in all cases with transactive response DNA-binding protein (TDP) proteinopathy type A, as expected. Additionally, most of the cases presented also with primary age-related tauopathy (PART) or mild Alzheimer’s disease (AD) neuropathological changes, and one case had extensive Lewy body pathology. An additional finding was the presence of cerebral small vessel changes in every patient in this family.

**Conclusions:**

Our data show not only that the IVS1 + 5G > C mutation has an exclusive association with FTLD-TDP type A proteinopathy but also that other proteinopathies can occur and should be looked for. Because the penetrance rate of the clinical phenotype of carriers of *GRN* mutations is age-dependent, further research is required to investigate the role of co-occurring age-related pathologies such as AD, PART, and cerebral small vessel disease.

**Electronic supplementary material:**

The online version of this article (doi:10.1186/s13195-017-0334-y) contains supplementary material, which is available to authorized users.

## Background

Frontotemporal lobar degeneration (FTLD) accounts for 10–15% of all dementias and represents the second most common cause of young-onset dementia. FTLD is always associated with atrophy of the frontal and/or temporal lobes and is sometimes markedly asymmetrical. The clinical phenotype of FTLD includes two major cognitive syndromes: behavioral variant frontotemporal dementia (bvFTD) and primary progressive aphasia (PPA) [1–3]. FTLD is often associated with extrapyramidal symptomatology, being referred to as *frontotemporal lobar degeneration with parkinsonism* (FTDP), or with motor neuron disorders (FTLD-MNDs), of which amyotrophic lateral sclerosis (ALS) is the most common.

A familial history is present in up to 40% of patients with FTLD, with a pattern suggestive of autosomal dominant inheritance in 10–20% of FTLD families. In 1998, frontotemporal dementia associated with parkinsonism was linked to chromosome 17 (FTDP-17), leading to the discovery of the microtubule-associated protein tau gene (*MAPT*) [4–6] in 1998 and of the progranulin gene (*GRN*) in 2006 [7, 8].

Mutations in *MAPT* are often associated with tau aggregates (FTLD-tau), whereas transactive response DNA-binding protein 43 (TDP-43) inclusions (FTLD-TDP) are found in patients with mutations in the *C9orf72* [9–11], *GRN* [7, 12], valosin-containing protein gene (*VCP*) [13], *TARDBP* [14], or *TBK1* genes [15]. Rare proteinopathies include FTLD-UPS (ubiquitin proteasome system), caused by mutations in chromatin-modifying protein 2B (*CHMP2B*) [16, 17] and FTLD-FUS (fused in sarcoma) [18], or FTLD with inclusions belonging to other proteins of the FET family (FUS, EWSR1, and TAF15 family of proteins) [19], for which the underlying genetic causes are still unknown.

TDP proteinopathies are classified into four subtypes, A through D [20]. TDP type A is characterized by many neuronal intracytoplasmic inclusions (NCIs) and short dystrophic neurites (DNs) predominantly in the second cortical layer, whereas TDP type B is represented by a moderate amount of NCIs and few DNs, present in all cortical layers. Type C inclusions are typically long DNs, mainly in the second cortical layer. TDP type D inclusions are characterized by many neuronal intranuclear inclusions (NIIs). Because the neuropathological diagnosis has a variable link with the clinical picture, the connection with the causative gene mutations is much stronger. *GRN* mutations are associated with FTLD-TDP type A. Mutations in *GRN* cause 15–40% of FTLD-TDP cases [21–24]. In Belgium, *GRN* mutations are the second most prevalent mutations causing FTLD, with *C9orf72* G_4_C_2_ repeat expansions being the most prevalent [25].

The penetrance rate seems to be age-dependent, just as it is in *C9orf72* mutations [25]. Gass et al. found that by the age of 60, nearly 50% of the carriers were affected, whereas 90% of patients showed symptomatology by the age of 90 [22]. The heterogeneity of the clinical phenotypes in *GRN* mutations and the incomplete penetrance of a mutation [26] can make it difficult to recognize an autosomal dominant pattern of inheritance.

*GRN* is located on chromosome 17q21, and the first loss-of-function mutations in *GRN* were identified in 2006 [7, 8]. Cruts et al. described a point mutation in the splice donor site of intron 1 (IVS1 + 5G > C) in the Belgian DR8 family [8, 12]. Consequently, intron 1 splicing is prohibited, and the mutant transcript is retained in the nucleus, where it is degraded, leading to a null allele and 50% of *GRN* production. This mutation was shown to be a founder mutation and is the most frequent *GRN* mutation in patients with frontotemporal dementia (FTD) in the Belgian Flanders population [8] (Wauters E, Van Mossevelde S, Sleegers K: Phenotypic characteristics and genetic onset age modifiers in an extended Belgian GRN founder family. Submitted). Since the identification of *GRN*, 172 different mutations have been described (http://www.molgen.ua.ac.be) [27]. Next to null mutations [28], partial deletions of the *GRN* gene have also been described in FTLD [29, 30]. GRN missense mutations have also been observed in Alzheimer’s disease (AD) and ALS [31–33].

Progranulin is a growth factor expressed by many cells, including neurons. Progranulin can be cleaved to form smaller peptides, called *granulins*, which have an additional role in inflammation, wound repair, and cell cycling [24]. As a result of the haploinsufficiency, low progranulin levels are found in the plasma, serum, and cerebrospinal fluid of symptomatic and asymptomatic *GRN* mutation carriers and were also present in the family we are presently reporting [34, 35].

Cerebral small vessel disease (SVD) is a common finding in the elderly brain, and its pathology appears in the presence of risk factors such as smoking habit, hypertension, hypercholesterolemia, and diabetes mellitus. There is a known link with AD and Lewy body disease [36], but to date there are no data regarding SVD in patients carrying a *GRN* mutation. De Reuck et al. found no cerebrovascular changes in 22 patients with FTLD, 2 of whom carried a *GRN* mutation [37]. Because Thal et al. recently found an association between cerebral SVD and Pick’s disease [38], it is of interest whether cerebral SVD could play a role in this disease.

We characterized the neuropathological data of nine members of the Belgian *GRN* founder family in an extensive sampling of brain regions, and we compared our data with the published clinical and neuropathological characteristics of other *GRN* mutations and other FTLD syndromes. Furthermore, because white matter pathology is a common finding on cerebral structural imaging of *GRN* mutation carriers, and because this finding was also observed in our *GRN* founder family, we examined the white matter and cerebral small vessels in different brain regions in this family.

## Methods

Neuropathological evaluation was carried out in nine members of an extended family of patients with FTLD carrying the *GRN* IVS1 + 5G > C mutation, which was previously reported in 2006 [8]. Neuropathological findings of one patient in this family (DR205.1) were previously described with the techniques available at that time. The nine patients were members of six different branches of the *GRN* founder family (for pedigree, *see* (Wauters E, Van Mossevelde S, Sleegers K: Phenotypic characteristics and genetic onset age modifiers in an extended Belgian GRN founder family. Submitted)). All available clinical and iconographical data of these patients were reviewed. Because the subjects originated from different regions in Flanders (Belgium), they were followed by different neurologists in different centers. Consequently, the diagnostic approaches and performed investigations or neuropsychological tests done during the patients’ lifetimes varied between patients and in the clinical data. For most of the patients, we had access to clinical data concerning medical history, clinical neurological evaluations, neuropsychological evaluations, and brain imaging (structural and functional). The right hemisphere was neuropathologically examined, as was the circle of Willis when present.

We used standardized sampling and staining techniques (*see* Additional file 1) to identify histological changes, proteinopathies, and cerebral small vessel pathology. For AD neuropathological diagnosis and for its clinicopathological correlation, the criteria of Montine et al. were used [39]; for Lewy body pathology, the classification of McKeith et al. was used [40]; and for TDP proteinopathies, we used the Mackenzie et al. criteria [20]. For staging of cerebrovascular pathology, we refer to the work of Deramecourt et al. [36], and for the clinicopathological correlations of the cerebrovascular pathology, we refer to the work by Skrobot et al. [41]. The methodological data concerning DNA extraction, clinical findings, neuropsychological assessments, and imaging can be found in Additional file 1.

## Results

### Neuropathological findings

#### Macroscopic findings

**Fig. 1:**
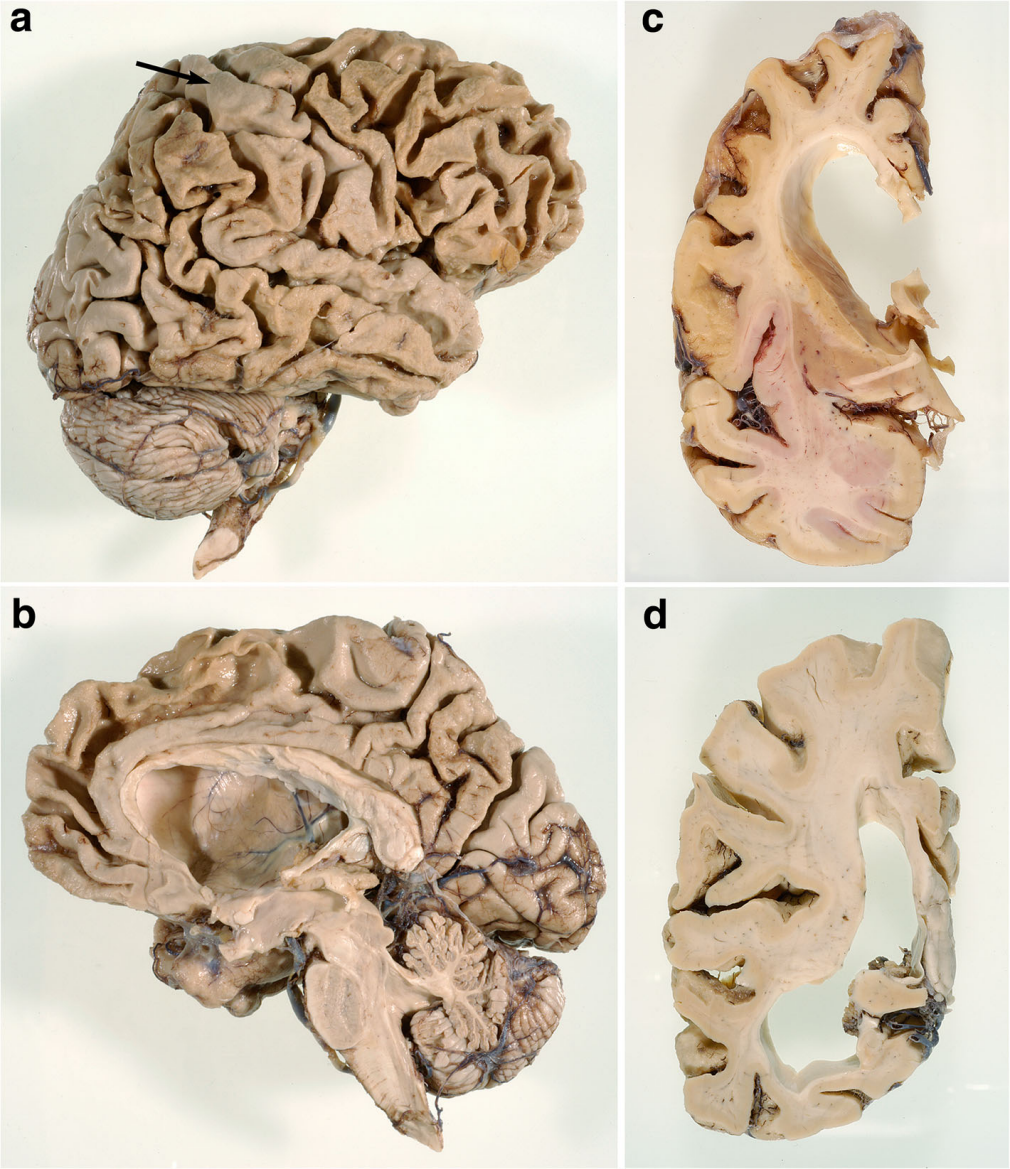
Macroscopic images. **a** Lateral view of the right hemisphere in DR2.3. Note the frontal and temporal, and to a lesser extent parietal, atrophy. Area 4 (*arrow*) is relatively preserved. **b** Medial view of right hemisphere in DR2.3. The superior frontal gyrus is atrophied, whereas the straight gyrus is relatively spared. **c** Coronal section of the right hemisphere trough the head of the caudate nucleus in DR2.3. There is a flattened, nearly concave aspect of the caudate nucleus. **d** Coronal sections trough the parietotemporal lobes in DR8.1. The lateral ventricle is dilated, especially the temporal horn. Temporal atrophy is more pronounced than parietal atrophy

Macroscopic findings in eight of nine patients (no data available for patient DR205.1) were compatible with moderate to severe frontal atrophy, with a relative sparing of the precentral gyrus (Fig. 1a, b; Table 1). To a lesser extent, temporal and/or parietal cortices (Fig. 1d) were also atrophied, in addition to the head of the caudate nucleus (Fig. 1c). For the full macroscopic data, *see* Additional file 2. Because our research protocol included the preservation of the left hemisphere at −80 °C, statements about asymmetry were not possible.

**Table 1:** Macroscopic findings

Atrophy location	DR2.3	DR8.1	DR25.1	DR25.5	DR28.1	DR205.1	DR31.1	DR1207.1	DR1213.1
Frontal lobe	+++	+++	+++	+++	+++	NA	+	+++	+++
Temporal lobe	+++	+	+	+++	+	NA	+	++	+
Parietal lobe	+	−	++	−	+++	NA	−	−	−
Caudate nucleus	+	+	NA	+	+	NA	+	+++	+++
Whole cortical atrophy	+	−	−	−	−	NA	−	−	−
Weight of right hemisphere	414 g	519 g	449 g	472 g	583 g	NA	NA	522 g	577 g

*NA* Not available, + Mild atrophy, ++ Moderate atrophy, +++ Severe atrophy

In three of six patients, we saw mild atherosclerotic plaques or minimal fibrotic changes in the arteries of the circle of Willis.

#### Histological findings

**Fig. 2:**
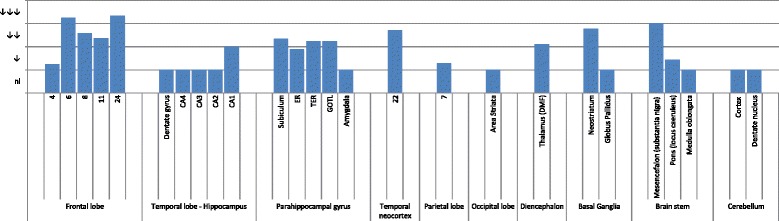
Grouped histopathological findings. Entorhinal cortex (ER), transentorhinal cortex (TER), gyrus occipitotemporalis lateralis (GOTL), dorsomedial formation (DMF), and status pigmentatus (SP). Semiquantitative analysis of the average neuronal cell loss in every region of interest. *nl* Normal, ↓ Slight neuronal loss, ↓↓ Moderate neuronal loss, ↓↓↓ Severe neuronal loss

In all patients, a moderate to severe cortical neuronal loss with astrocytosis was seen in Brodmann area 24, more than in areas 6–8 and 11 and more than in precentral area 4. The temporal neocortex was atrophic in three cases, whereas every case presented with mild to moderate neuronal loss in CA1, the subiculum, and the pro- and parasubiculum (Table 2). There was severe atrophy in the neostriatum; the substantia nigra had substantial neuronal loss in all cases, whereas the locus coeruleus was affected in four of nine patients. There were no abnormalities in the cerebellum or thalamus. Figure 2 summarizes the major histological abnormalities.

**Table 2:** Co-occurring Alzheimer’s disease pathology and clinicopathological correlation, hippocampal neuronal loss, and apolipoprotein E genotype

Identifier	Montine	Thal	Braak	CERAD	Correlation	Hippocampal neuronal loss	ApoE genotype
DR2.3	A1B1C1	2	2	1	Low	2	Ɛ2/Ɛ3
DR8.1	A0B1C0	0	1	0	Not	2	Ɛ3/Ɛ3
DR25.1	A0B1C0	0	1	0	Not	1	Ɛ3/Ɛ3
DR25.5	A0B1C0	0	1	0	Not	2	Ɛ3/Ɛ3
DR28.1	A3B1C1	5	1	1	Low	0	Ɛ3/Ɛ4
DR205.1	A2B1C1	3	2	1	Low	1	Ɛ2/Ɛ4
DR31.1	A1B1C1	2	1	1	Low	1	Ɛ2/Ɛ3
DR1207.1	A0B1C0	0	1	0	Not	1	Ɛ3/Ɛ3
DR1213.1	A0B1C0	0	1	0	Not	2	Ɛ3/Ɛ3

*ApoE* Apolipoprotein E, *CERAD* Consortium to Establish a Registry for Alzheimer’s Disease

Column 2: Montine classification [39, 83]; column 3: Thal stages for β-amyloid plaques [84]; column 4: Braak stages for neurofibrillary changes [85]; column 5: CERAD stages for senile plaques [86]: 0 = no pathology, 1 = sparse CERAD score; column 6: clinicopathological correlation of Alzheimer’s disease neuropathological changes [39]; column 7: neuronal loss in hippocampal CA1 (scale 0–3): 0 = no pathology, 1 = mild neuronal loss, 2 = moderate neuronal loss; column 8: ApoE genotype

#### Small vessel disease

**Fig. 3:**
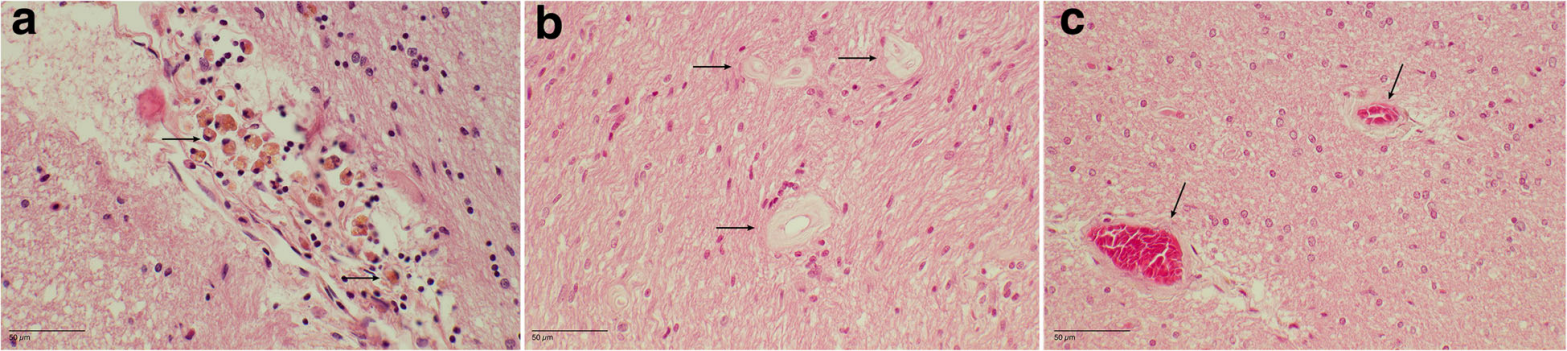
Cerebral small vessel disease. **a** DR2.3. H&E stain of perivascular hemosiderin deposits in the hippocampus. *Arrows* show many siderophages. **b** DR25.5. H&E stain of the corpus callosum. *Arrows* show thickening of the arteriolar wall. **c** DR25.5. H&E stain of the temporal white matter. *Arrows* show thickening of the arteriolar walls with mild perivascular demyelination

In every patient, small vessel changes were present. Trichrome staining showed a preferential thickening of the arteriolar wall in cortex and white matter. (Small arteries were defined as arterioles when the diameter was between 40 and 150 μm [36].) Arteriolosclerosis was characterized by thickening of the vessel wall and reduplication of the internal elastic layer. Other features of SVD included a dilation of the perivascular space, perivascular demyelination, and perivascular hemosiderin leakage (Fig. 3). The staging system of Deramecourt et al. [36] was used to semiquantify SVD. In the frontal lobes, SVD was more severe than in the temporal lobes, basal ganglia, and hippocampus (Table 3). Using the vascular cognitive impairment neuropathological guidelines, on the basis of the presence of leptomeningeal cerebral amyloid angiopathy, subcortical macroinfarcts, and arteriolosclerosis in the occipital lobe, we could estimate that there was a low likelihood that the cerebrovascular pathology contributed to the cognitive decline in our cases (Table 3).

**Table 3:** Cerebral small vessel disease and its clinicopathological correlation

Identifier	Frontal	Temporal	Hippocampal	Basal ganglia	Total score	CAA	Large infarcts	Arteriolosclerosis	Correlation
DR2.3	1	1	1	2	5	0	0	0	Low
DR8.1	2	2	2	1	7	1	0	1	Low
DR25.1	2	1	2	1	6	0	0	0	Low
DR25.5	2	2	1	1	6	0	0	2	Low
DR28.1	1	1	1	1	4	0	0	1	Low
DR205.1	NA	NA	2	1	NA	NA	NA	NA	Low
DR31.1	2	1	1	1	5	0	0	1	Low
DR1207.1	2	1	1	1	5	0	0	1	Low
DR1213.1	1	1	1	2	5	0	0	0	Low

*CAA* Leptomeningeal cerebral amyloid angiopathy, *NA* Not applicable

Deramecourt staging of cerebrovascular pathology in dementia in frontal lobe, temporal lobe, and hippocampus, with total score (total possible score = 20) [36]. For the likelihood that cerebral small vessel disease (SVD) contributed to cognitive decline, we refer to the work of Skrobot et al. [41]. Large infarcts: one or more subcortical infarcts with diameter > 10 mm; arteriolosclerosis: arteriolosclerosis in the occipital lobe. Correlation: likelihood that SVD contributed to cognitive decline

#### Immunohistology

**Fig. 4:**
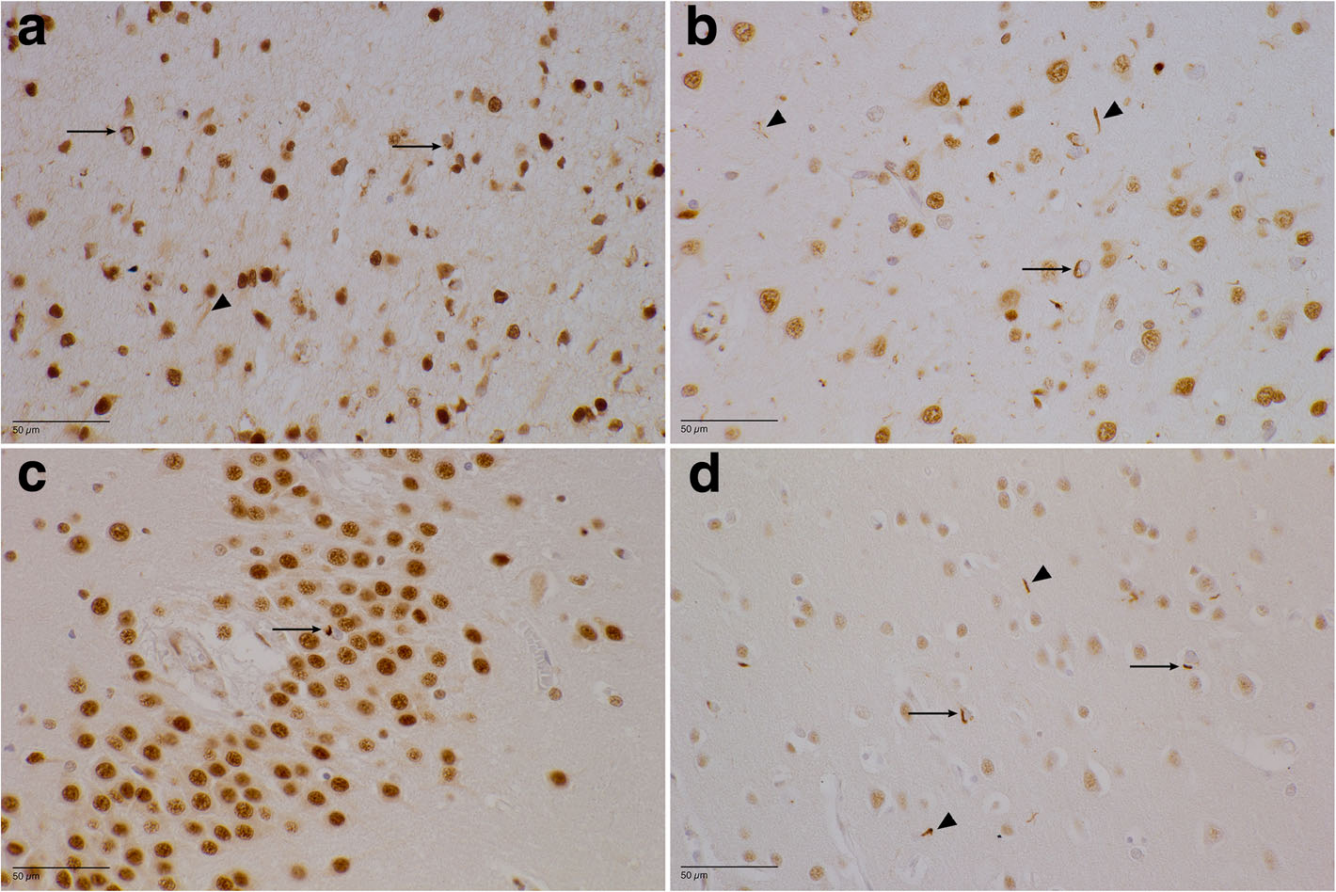
Transactive response DNA-binding protein (TDP) pathology (paraffin-embedded sections stained with antihyperphosphorylated TDP-43 antibody). **a** DR25.5 area 6. There is a moderate amount of neuronal intracytoplasmic inclusions (NCIs) (*arrows*), mainly in the second cortical layer. The dystrophic neurites (*arrowhead*) are more evenly spread throughout the entire cortex. **b** DR2.3 superior temporal gyrus. Mild to moderate TDP-43 proteinopathy type A with NCIs (*arrow*) and dystrophic neurites (*arrowheads*) can be seen. **c** DR28.1 dentate gyrus. There are scarce NCIs (*arrow*) in the granular layer. **d** DR31.1 area striata. A mild amount of NCIs (*arrows*) and dystrophic neurites (*arrowheads*) in the second cortical layer can be seen

In all patients, TDP proteinopathy type A could be diagnosed (Fig. 4). Lesions were, as expected, more severe in the frontal and temporal neocortices. In the frontal cortex, most lesions were found in areas 6 and 8, area 24, and area 11, compared with prefrontal area 4. The temporal superior gyrus demonstrated a moderate to high lesion load, whereas only a mild to moderate amount of TDP pathology was present in the hippocampal and parahippocampal structures.

In eight patients (no data available for DR205.1), there was mild to moderate TDP-43 proteinopathy in the parietal cortex (area 7), whereas in four of eight patients, the occipital lobe was affected. In the neostriatum, lesions were moderate to numerous and were evenly spread throughout the caudate nucleus and putamen. Sparse lesions were found in the dorsomedial formation of the thalamus in seven of nine patients.

Glial cytoplasmic inclusions (GCIs) were found in the subcortical and deeper white matter of the frontal and parietal lobes and rarely in the mesotemporal white matter. Except for rare DNs and occasional GCIs, no inclusions were found in the substantia nigra, pons, or medulla oblongata. No NCIs were present in the neurons of the hypoglossal nucleus. The cerebellum was normal.

**Fig. 5:**
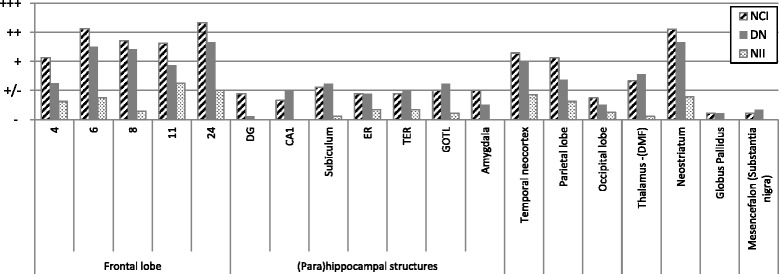
Transactive response DNA-binding protein pathology in different brain areas. ± (sparse (0–1/mm^2^)), + (mild (< 5/mm^2^)), ++ (moderate (5–20/mm^2^)), +++ (extensive (> 20/mm)). The average lesion load is shown for the most important regions of interest. Striped bar represents the amount of neuronal intracytoplasmic inclusions, whereas the dark bar represents the amount of short dystrophic neurites. With the dotted bar, the neuronal intranuclear inclusions are shown. *NCI* Neuronal intracytoplasmic inclusions, *DN* Dystrophic neurites, *NII* Nuclear intraneuronal inclusions, *DMF* Dorsomedial formation, *ER* Entorhinal cortex, *TER* Transentorhinal cortex, *GOTL* Gyrus occipitotemporalis lateralis, *DG* Dentate gyrus

The degree of TDP-43 inclusions was moderate, varying from sparse (0–1/mm^2^) (±), to mild (<5/mm^2^) (+), to moderate (5–20/mm^2^) (++), to extensive (> 20/mm^2^) (+++). The most common lesions were skein-like NCIs and short DNs. Sporadically, cat’s-eye NIIs were found, mainly in the frontotemporal neocortex. The lesion load was most profound in the second cortical layer. A summary of the TDP pathology is presented in Fig. 5.

In all patients, FUS and prion staining was negative. P62 stain could not elicit any additional pathology. In all patients, AD-related pathology was found; however, the lesion load remained mild. Two patients had A1B1C1 (DR2.3, DR31.1), one patient presented with A2B1C1 (DR205.1), and one patient presented with A3B1C1 (DR28.1). The five remaining patients presented with neurofibrillary tangles in the hippocampus and parahippocampal gyrus without β-amyloid pathology, diagnosed as AD neuropathological changes A0B1C0 [39], also referred to as *primary age-related tauopathy* (PART) [42]. Table 2 summarizes the AD neuropathological changes using the Montine criteria, as well as the clinicopathological correlation.

One patient (DR205.1) clinically presented with a combination of FTLD and Parkinson’s disease (PD). This case revealed many Lewy bodies in the substantia nigra and the formatio reticularis, and to a lesser extent in the frontal and temporal cortices, the hippocampus, and the parahippocampal gyrus. A dual diagnosis of FTLD-TDP type A and diffuse Lewy body disease, neocortical type, was made [40].

### Clinical, neuropsychological, and imaging data

We evaluated nine patients (four females, five males). Symptomatology started between the ages of 55 and 70 years, with a mean onset age of 62. The disease lasted, on average, 5.3 years (range 2.3–8.6). The mean age at death was 67 years (range 60–75). Symptomatology was mostly frontotemporal (four of nine bvFTD, four of nine PPA), except in one patient who was clinically diagnosed with PD (DR205.1). This patient developed frontotemporal symptoms as well, later in the disease. The demographic characteristics of the evaluated patients are summarized in Table 4, whereas the clinical and neuropsychological characteristics, as well as imaging data, are discussed in Additional files 3 and 4. Table 5 summarizes the cerebrovascular risk factors.

**Table 4:** Demographic data

Patient	Sex	FH	AO (years)	Age of death (years)	DD (years)	Final clinical diagnosis
DR2.3	F	F - autosomal dominant	63	72	8.6	PPA (NFV)
DR8.1	F	F - autosomal dominant	62	68	5.9	bvFTD
DR25.1	F	F - autosomal dominant	69	75	5.4	bvFTD
DR25.5	M	F - autosomal dominant	70	73	3,4	bvFTD
DR28.1	M	F - autosomal dominant	56	62	6.8	PPA (NFV)
DR205.1	F	F	55	61	5.1	PD
DR31.1	M	F	65	70	5.6	PPA (NFV)
DR1207.1	F	S	62	66	4.4	PPA (NFV)
DR1213.1	M	F - autosomal dominant	58	60	2.3	bvFTD

*Abbreviations: FH* Familial history), *F* Familial presentation, *S* Sporadic, *AO* Age of onset, *DD* Disease duration in years, *PPA* Primary progressive aphasia, *NFV* Nonfluent variant, *bvFTD* Behavioral variant frontotemporal dementia

**Table 5:** Cerebrovascular risk factors

DR	Smoking	Diabetes mellitus	Arterial hypertension	Hypercholesterolemia (> 190 mg/dl)	Other
DR2.3	Y (35 py)	Y	Y	N	
DR8.1	N	N	Y	Y	TIA
DR25.1	?	N	Y	N	Obesity
DR25.5	Y (40 py)	N	?	?	TIA, stroke
DR28.1	?	N	N	Y	
DR205.1	?	N	N	N	
DR31.1	?	?	?	Y	
DR1207.1	N	N	N	Y	
DR1213.1	N	N	N	N	

*Abbreviations: Y* Present, *N* Absent, *?* Unknown, *py* Pack-years, *TIA* Transient ischemic attack

## Discussion

This report describes the clinical and neuropathological findings in nine members of a Belgian *GRN* founder family. This extended Belgian founder family was first described by van der Zee et al. in 2006 after the identification of an ancestral haplotype at 17q21 in seven (unrelated) familial tau-negative patients with FTLD. The IVS1 + 5G > C *GRN* mutation was found as a causal gene defect [8]. This family has been further explored, and to date we have genealogical data of 29 branches over 5 generations.

### Neuropathology

Severe frontal and temporal neocortical loss was observed. Next to the neocortical atrophy, every patient had a neuronal cell loss in CA1 and the subiculum. There was no hippocampal sclerosis. These findings are comparable with the data published by Josephs et al. in 2007 [43]. In all our cases, TDP-43 type A proteinopathy was observed, confirming previously published data [20].

We compared our neuropathological data in these patients with data of patients in our database carrying other *GRN* mutations (*n* = 2), *C9orf72* expansion repeat mutations (*n* = 15), *TBK1* mutations (*n* = 2), and *VCP* mutations (*n* = 3) [15, 25, 44, 45]. In our study, the presence of a TDP type A proteinopathy and the absence of dipeptide repeat pathology in the dentate gyrus and hippocampus allowed us to differentiate between *GRN* and *C9orf72* mutations. We found no differences in TDP lesion load between the IVS1 + 5G > C *GRN* mutation and other *GRN* mutations. We observed that, compared with the *C9orf72* carriers, the precentral cortex area 4 was less affected. In both *GRN* and *C9orf72* carriers, parietal involvement was present; however, it was less explicit than frontotemporal atrophy. In four patients carrying the IVS1 + 5G > C *GRN* mutation, we also found a mild TDP proteinopathy in the occipital cortex.

In the *TBK1* carriers, the neuropathological diagnosis was compatible with a TDP type B proteinopathy, with involvement of all the frontal and temporal neocortices, the hippocampal dentate gyrus, the parahippocampal transentorhinal cortex, the neostriatum, and the pallidum. TDP pathology was also present in motor neurons, whereas in the *GRN* patients, this was not the case. The *VCP* patients carried an abundant load of TDP pathology in similar affected areas. The main difference was the high amount of intranuclear inclusions, compatible with a TDP proteinopathy type D.

We can assume that the clinical presentation is not linked to the TDP proteinopathy itself, but rather is the consequence of the specific cortical areas that are involved. The finding of TDP type A pathology spread throughout the entire cortex, though to a lesser extent in the parietal and occipital cortices, can explain the generalized cortical brain atrophy visualized by structural imaging [46, 47]. No TDP-43 pathology was found in the hypoglossal nucleus in our study, supporting the clinical evidence that motor neuron disease was not present in this family.

Because TDP-43 inclusions can also be found in carriers of *VCP* and *C9orf72* mutations, this suggests that TDP-43 dysregulation is merely a downstream effect through a common pathway leading to cell death [48]. The finding of the TDP inclusions is indeed not specific for underlying gene defects, although the pattern of TDP inclusions and associated proteinopathies can be suggestive of a specific gene mutation.

The distribution of the TDP-43 pathology in specific neocortical structures favors a protein distribution through neuronal pathways instead of proximity [49]. It is assumed that every specific neurodegenerative syndrome has its own specific region working as an “epicenter.” This epicenter is critical in the neuronal networks, whose normal connectivity profiles resemble the pattern of atrophy in that specific disease, in our case FTLD. Several network degeneration mechanisms have been suggested [50]. The presence of neostriatal lesions could support this hypothesis of degeneration of neuronal networks because the striatum has projections to many (frontotemporal) cortical areas. In addition to cortical abnormalities, white matter involvement visualized by cerebral imaging has been reported in patients carrying a *GRN* mutation [21, 51, 52]. Compared with patients with *MAPT* mutations, however, *GRN* mutation carriers would tend to show less white matter abnormalities [53]. Because white matter pathology was also seen on neuroimaging studies in five of our patients, we investigated the proteinopathy and vessel involvement in the cortex and white matter. Glial TDP proteinopathy is regularly found in patients with *GRN* mutations, and this was also the case in all our patients [53].

Furthermore, we found mild to moderate cerebral SVD, most pronounced in the frontal lobe, whereas only normal vascular findings have been described in patients with FTLD [37, 54]. We assume that the SVD is merely the consequence of the age of our patients as well as the comorbidity and presence of cerebrovascular risk factors. The clinicopathological correlation of the cerebral vascular pathology is low, but an influence on the disease mechanism cannot be excluded.

Additionally, a concomitant neuropathological diagnosis of AD or PART could be made in all patients. In every case, however, neuropathological findings suggestive of AD were mild and could be classified as not more than A3B1C1. As presented in Table 2, we assume that there is no correlation between the neuropathological findings and the clinical symptomatology. Five patients could be considered as examples of PART [40]. One patient presented with a mixed diagnosis of FTLD-TDP and diffuse Lewy body pathology, neocortical type.

We investigated whether the apolipoprotein E (ApoE) genotype (Table 2) could be related to the AD pathology. Most patients were homozygous for the ApoE ɛ3 allele (five of nine). Three patients were carrying the ApoE ɛ2 allele, whereas two patients were heterozygous carriers of the ApoE ɛ4 risk allele. These latter two patients had a slightly more severe Montine score for AD pathology, but without clinical significance.

The high frequency of concomitant pathologies in *GRN* mutation carriers, such as the combination of FTLD with PD or FTLD with AD [55–57], might indicate that loss or partial loss of *GRN* could advance pathological features of other neurodegenerative diseases in an early stage [58]. Petkau and Leavitt reviewed data on progranulin in neurodegenerative diseases and suggested that *GRN* variants resulting in decreased progranulin expression might be risk factors for both FTLD and other dementias and that common *GRN* variants can act as modifying factors for age of onset, disease duration, and risk of disease in ALS, AD, multiple sclerosis, bipolar disorder, and schizophrenia [59].

The combined occurrence of TDP-43 lesions, AD pathology, and PD pathology favors the hypothesis that progranulin is a protective growth factor, with the IVS1 + 5G > C mutation resulting in a loss of function of progranulin. This results from a cascade of defective biological processes in an expansion of neurodegenerative processes leading toward protein deposits and cell degeneration [60]. However, because AD and PD are frequent neurodegenerative disorders, a coexistence of these disorders could be merely a consequence of age-related risk factors. Different studies have elicited the co-occurrence of proteinopathies in patients carrying a *GRN* mutation [21, 43, 54], whereas other groups could not confirm this [43].

### Clinical presentation

Our clinical data confirm that the age of disease onset is older than in patients with *MAPT* or *C9orf72* mutations. Symptomatology started at the age of 62 (range 55–70), and the disease lasted, on average, 5.3 years (2.30–8.56), whereas disease in *MAPT* mutation carriers often starts earlier. Van Langenhove et al. [25] found a mean onset age of 56.9 years (range 49–65) in a Belgian FTLD cohort with the *MAPT* mutation, and Whitwell et al. [61] described an even younger mean onset age of 44 years (range 24–63) in *MAPT* mutation carriers. In patients with a *C9orf72* repeat expansion, the described mean onset age was younger as well. Van Langenhove et al. [25], and later Van Mossevelde et al. [62], described mean onset ages of 55.3 years (range 42–69) and 54.3 years (range 29–75), respectively, in Belgian patients with FTLD with a *C9orf72* repeat expansion.

When we compare our findings with those in other *GRN* mutation carriers, the findings are similar, although a broad range of onset ages has been described. In the whole Belgian *GRN* founder family, onset ages varying between 45 and 70 years have been described [8]. Different cohorts of *GRN* mutation carriers have resulted in different mean onset ages with broad ranges from 55 years (range 37–72) [63] to 64.5 years (range 49–88) [64]. Even within one family of *GRN* mutation carriers, broad ranges have been described for the onset age. Snowden et al. described a variation in the onset ages in one family of 23 years [57]. These highly variable onset ages indicate that there are other modifying factors that modulate onset age in *GRN* mutation carriers. *GRN* mutations are most frequently associated with bvFTD followed by PPA [21].

In our four patients with a diagnosis of bvFTD, apathy was one of the initial predominant symptoms. This observation is in line with previous findings that apathy dominates the clinical picture in patients with bvFTD caused by a *GRN* mutation [65, 66]. It is in contrast with bvFTD caused by *C9orf72* repeat expansion, in which inappropriate behavior and agitation dominate the clinical presentation [25]. Also, the fact that four of the nine family members had PPA is highly suggestive for a *GRN* mutation [21]. The PPA phenotype in *GRN* mutation carriers is most often distinct from that subsumed under the classical PPA subtypes [67].

Other phenotypes have been associated with *GRN* mutations and include AD [68], PD [65], corticobasal syndrome [69–71], dementia with Lewy bodies [72], and mild cognitive impairment [73]. Also, several psychiatric syndromes have been associated with *GRN* mutations [74, 75]. MND is very rarely described in association with *GRN* mutations [24, 76]. None of our nine patients had a concomitant MND. There was one co-occurrence with PD. Parkinsonism occurs quite frequently in patients with *GRN* mutation, but usually later in the disease course, after development of FTD. In a few cases, parkinsonism was described as the first or predominant clinical manifestation of a *GRN* mutation. In a cohort of 32 French *GRN* mutation carriers, 13 (41%) developed parkinsonism during their disease course, but it was present at onset in only 1 patient [77]. Kelley et al. studied patients with FTLD-UPS and found that parkinsonism was an initial/early symptom in 3 of 18 patients with a *GRN* mutation [64]. Although parkinsonism occurs quite frequently in patients with FTLD with a *GRN* mutation, *GRN* genetic variability is unlikely to contribute significantly to susceptibility to PD [58].

Six of our patients had subjective or objective signs of memory loss early or later in the disease course. All of our patients had mild AD neuropathological features or PART, but without strong clinicopathological correlation.

Le Ber et al. [77] described that clinical AD features such as episodic memory disorders are frequent in *GRN* mutation carriers (89%). Of the 32 *GRN* mutation carriers evaluated by Le Ber et al., 3 were clinically diagnosed with AD because of predominant episodic memory disorder. In the study of Chen-Plotkin et al., 11 of 97 (11.3%) TDP-positive *GRN* mutation carriers received a clinical diagnosis of AD [24].

Neuropsychological evaluation showed extensive frontal executive dysfunction in six of seven patients. Neocortical temporal deficits (economy of speech, comprehension deficits, naming deficits) were present in all patients. Apraxia syndromes were seen in three patients, suggesting parietal involvement.

### Imaging

Cerebral structural imaging demonstrated atrophy in all 9 patients, mostly asymmetric at the frontal and temporal lobes and often with a parietal cortical involvement. Similar findings were observed by functional neuroimaging, where hypoperfusion and hypometabolism were asymmetric (left versus right), most explicit in the frontal and temporal lobes. Again, parietal involvement often was present.

These findings confirm the previously published data in which parietal involvement [61, 78, 79] and marked asymmetry of atrophy/hypofunction were suggested as distinctive features of *GRN* mutations [46, 77, 79, 80], as well as a relatively fast evolution to generalized brain atrophy [47]. Seven patients had marked asymmetry, and in five of them, the most pronounced atrophy/hypofunction was present in the left hemisphere. There seems to be no preferential side of predominant atrophy/hypofunction in *GRN* mutation carriers. Previous studies have demonstrated predominant left-side involvement [55, 57] as well as predominant right-side involvement [77, 78, 81, 82].

## Conclusions

This study is the first, to our knowledge, in which nine members of one family carrying a *GRN* mutation have undergone exhaustive neuropathological analysis. Our study made it possible to link this specific *GRN* mutation to TDP-43 proteinopathy subtype A.

The neocortical pathology in all our patients was very explicit, with impairment of frontal and temporal cortices to the greatest extent, followed by parietal and, to a lesser extent, occipital involvement. In all of our cases, mild hippocampal atrophy was present, but without hippocampal sclerosis. Next to the TDP-43 proteinopathy subtype A, we found the co-occurrence of other proteinopathies, such as PART in five cases, mild AD in four cases, and Lewy body disease in one case, and we demonstrated the coexistence of small vessel changes, most explicit in the frontal lobe of every patient. We do not think that the small vessel pathology is associated with the *GRN* mutation, but an age-dependent penetrance rate has been demonstrated in *GRN* carriers. Whether the clinical presentation is the result of the TDP pathology exceeding a critical limit of neuronal damage or of the co-occurrence of age-related AD pathology or PART or SVD should be further investigated. Because the age of onset in these patients lies between 55 and 70 years, an age range within which cerebrovascular risk factors have their impact, it should be further assessed whether this cerebrovascular pathology has an influence on the clinical presentation, onset, and disease course of the proteinopathy.

## Additional files

Additional file 1: Materials and methods. (DOCX 26 kb)

Additional file 2: Results: neuropathology. (DOCX 22 kb)

Additional file 3: Results: clinical and neuropsychological assessment. (DOCX 24 kb)

Additional file 4: Results: imaging data. (DOCX 16 kb)

## Abbreviations

AD: Alzheimer’s disease
ALS: Amyotrophic lateral sclerosis
AO: Age of onset
APOE: Apolipoprotein E
bvFTD: Behavioral variant frontotemporal dementia
CAA: Leptomeningeal cerebral amyloid angiopathy
CERAD: Consortium to Establish a Registry for Alzheimer’s Disease
*CHMP2B*: Chromatin-modifying protein 2B
DD: Disease duration in years
DMF: Dorsomedial formation
DN: Dystrophic neurite
ER: Entorhinal cortex
F: Familial presentation
FET family: FUS, EWSR1, and TAF15 family of proteins
FH: Familial history
FTDP: Frontotemporal lobar degeneration with parkinsonism
FTDP-17: Frontotemporal dementia associated with parkinsonism linked to chromosome 17
FTLD: Frontotemporal lobar degeneration
FUS: Fused in sarcoma
GCI: Glial cytoplasmic inclusion
GOTL: Gyrus occipitotemporalis lateralis
*GRN*: Progranulin gene
*MAPT*: Microtubule-associated protein tau gene
MND: Motor neuron disorder
NCI: Neuronal intracytoplasmic inclusion
NFV: Nonfluent variant
NII: Neuronal intranuclear inclusion
nl: Normal
PART: Primary age-related tauopathy
PD: Parkinson’s disease
PPA: Primary progressive aphasia
py: Pack-year
SP: Status pigmentatus
SVD: Cerebral small vessel disease
TDP-43: Transactive response DNA-binding protein 43
TER: Transentorhinal cortex
TIA: Transient ischemic attack
UPS: Ubiquitin proteasome system
*VCP*: Valosin-containing protein gene

## References

[1] Rascovsky K (2011). Sensitivity of revised diagnostic criteria for the behavioural variant of frontotemporal dementia. Brain.

[2] Neary D (1998). Frontotemporal lobar degeneration: a consensus on clinical diagnostic criteria. Neurology.

[3] Gorno-Tempini ML (2011). Classification of primary progressive aphasia and its variants. Neurology.

[4] Hutton M (1998). Association of missense and 5′-splice-site mutations in *tau* with the inherited dementia FTDP-17. Nature.

[5] Poorkaj P (1998). Tau is a candidate gene for chromosome 17 frontotemporal dementia. Ann Neurol.

[6] Spillantini MG (1998). Mutation in the tau gene in familial multiple system tauopathy with presenile dementia. Proc Natl Acad Sci U S A.

[7] Baker M (2006). Mutations in progranulin cause tau-negative frontotemporal dementia linked to chromosome 17. Nature.

[8] Cruts M (2006). Null mutations in progranulin cause ubiquitin-positive frontotemporal dementia linked to chromosome 17q21. Nature.

[9] DeJesus-Hernandez M (2011). Expanded GGGGCC hexanucleotide repeat in noncoding region of *C9ORF72* causes chromosome 9p-linked FTD and ALS. Neuron.

[10] Gijselinck I (2012). A *C9orf72* promoter repeat expansion in a Flanders-Belgian cohort with disorders of the frontotemporal lobar degeneration-amyotrophic lateral sclerosis spectrum: a gene identification study. Lancet Neurol.

[11] Renton AE (2011). A hexanucleotide repeat expansion in *C9ORF72* is the cause of chromosome 9p21-linked ALS-FTD. Neuron.

[12] Cruts M, Kumar-Singh S, Van Broeckhoven C (2006). Progranulin mutations in ubiquitin-positive frontotemporal dementia linked to chromosome 17q21. Curr Alzheimer Res.

[13] Watts GD (2004). Inclusion body myopathy associated with Paget disease of bone and frontotemporal dementia is caused by mutant valosin-containing protein. Nat Genet.

[14] Sreedharan J (2008). TDP-43 mutations in familial and sporadic amyotrophic lateral sclerosis. Science.

[15] Gijselinck I (2015). Loss of *TBK1* is a frequent cause of frontotemporal dementia in a Belgian cohort. Neurology.

[16] Mackenzie IR (2011). Novel types of frontotemporal lobar degeneration: beyond tau and TDP-43. J Mol Neurosci.

[17] Skibinski G (2005). Mutations in the endosomal ESCRTIII-complex subunit CHMP2B in frontotemporal dementia. Nat Genet.

[18] Neumann M (2009). A new subtype of frontotemporal lobar degeneration with FUS pathology. Brain.

[19] Neumann M (2011). FET proteins TAF15 and EWS are selective markers that distinguish FTLD with FUS pathology from amyotrophic lateral sclerosis with *FUS* mutations. Brain.

[20] Mackenzie IR (2011). A harmonized classification system for FTLD-TDP pathology. Acta Neuropathol.

[21] van Swieten JC, Heutink P (2008). Mutations in progranulin (*GRN*) within the spectrum of clinical and pathological phenotypes of frontotemporal dementia. Lancet Neurol.

[22] Gass J (2006). Mutations in progranulin are a major cause of ubiquitin-positive frontotemporal lobar degeneration. Hum Mol Genet.

[23] Rohrer JD, Warren JD (2011). Phenotypic signatures of genetic frontotemporal dementia. Curr Opin Neurol.

[24] Chen-Plotkin AS (2011). Genetic and clinical features of progranulin-associated frontotemporal lobar degeneration. Arch Neurol.

[25] Van Langenhove T (2013). Distinct clinical characteristics of *C9orf72* expansion carriers compared with *GRN*, *MAPT*, and nonmutation carriers in a Flanders-Belgian FTLD cohort. JAMA Neurol.

[26] Cruts M, Van Broeckhoven C (2008). Loss of progranulin function in frontotemporal lobar degeneration. Trends Genet.

[27] Cruts M, Theuns J, Van Broeckhoven C (2012). Locus-specific mutation databases for neurodegenerative brain diseases. Hum Mutat.

[28] Gijselinck I (2008). Progranulin locus deletion in frontotemporal dementia. Hum Mutat.

[29] Clot F (2014). Partial deletions of the *GRN* gene are a cause of frontotemporal lobar degeneration. Neurogenetics.

[30] van der Zee J (2007). Mutations other than null mutations producing a pathogenic loss of progranulin in frontotemporal dementia. Hum Mutat.

[31] Gijselinck I, Van Broeckhoven C, Cruts M (2008). Granulin mutations associated with frontotemporal lobar degeneration and related disorders: an update. Hum Mutat.

[32] Sleegers K (2008). Progranulin genetic variability contributes to amyotrophic lateral sclerosis. Neurology.

[33] Brouwers N (2008). Genetic variability in progranulin contributes to risk for clinically diagnosed Alzheimer disease. Neurology.

[34] Sleegers K (2009). Serum biomarker for progranulin-associated frontotemporal lobar degeneration. Ann Neurol.

[35] Ghidoni R (2008). Low plasma progranulin levels predict progranulin mutations in frontotemporal lobar degeneration. Neurology.

[36] Deramecourt V (2012). Staging and natural history of cerebrovascular pathology in dementia. Neurology.

[37] De Reuck JL (2012). Cerebrovascular lesions in patients with frontotemporal lobar degeneration: a neuropathological study. Neurodegener Dis.

[38] Thal DR (2015). Frontotemporal lobar degeneration FTLD-tau: preclinical lesions, vascular, and Alzheimer-related co-pathologies. J Neural Transm.

[39] Montine TJ (2012). National Institute on Aging-Alzheimer’s Association guidelines for the neuropathologic assessment of Alzheimer’s disease: a practical approach. Acta Neuropathol.

[40] McKeith IG (2006). Consensus guidelines for the clinical and pathologic diagnosis of dementia with Lewy bodies (DLB): report of the Consortium on DLB International Workshop. J Alzheimers Dis.

[41] Skrobot OA (2016). Vascular cognitive impairment neuropathology guidelines (VCING): the contribution of cerebrovascular pathology to cognitive impairment. Brain.

[42] Duyckaerts C (2015). PART is part of Alzheimer disease. Acta Neuropathol.

[43] Josephs KA (2007). Neuropathologic features of frontotemporal lobar degeneration with ubiquitin-positive inclusions with progranulin gene (*PGRN*) mutations. J Neuropathol Exp Neurol.

[44] Bit-Ivan EN (2014). A novel *GRN* mutation (GRN c.708 + 6_ + 9delTGAG) in frontotemporal lobar degeneration with TDP-43-positive inclusions: clinicopathologic report of 6 cases. J Neuropathol Exp Neurol.

[45] van der Zee J (2009). Clinical heterogeneity in 3 unrelated families linked to *VCP* p.Arg159His. Neurology.

[46] Rohrer JD (2010). Distinct profiles of brain atrophy in frontotemporal lobar degeneration caused by progranulin and tau mutations. Neuroimage.

[47] Whitwell JL (2011). Trajectories of brain and hippocampal atrophy in FTD with mutations in *MAPT* or *GRN*. Neurology.

[48] Rademakers R, Neumann M, Mackenzie IR (2012). Advances in understanding the molecular basis of frontotemporal dementia. Nat Rev Neurol.

[49] Raj A, Kuceyeski A, Weiner M (2012). A network diffusion model of disease progression in dementia. Neuron.

[50] Zhou J (2012). Predicting regional neurodegeneration from the healthy brain functional connectome. Neuron.

[51] Caroppo P (2014). Extensive white matter involvement in patients with frontotemporal lobar degeneration: think progranulin. JAMA Neurol.

[52] Bozzali M (2013). Structural brain signature of FTLD driven by Granulin mutation. J Alzheimers Dis.

[53] McMillan CT (2013). White matter imaging helps dissociate tau from TDP-43 in frontotemporal lobar degeneration. J Neurol Neurosurg Psychiatry.

[54] Le Ber I (2007). Progranulin null mutations in both sporadic and familial frontotemporal dementia. Hum Mutat.

[55] Spina S (2007). Clinicopathologic features of frontotemporal dementia with progranulin sequence variation. Neurology.

[56] Rademakers R (2007). Phenotypic variability associated with progranulin haploinsufficiency in patients with the common 1477C → T (Arg493X) mutation: an international initiative. Lancet Neurol.

[57] Snowden JS (2006). Progranulin gene mutations associated with frontotemporal dementia and progressive non-fluent aphasia. Brain.

[58] Nuytemans K (2008). Progranulin variability has no major role in Parkinson disease genetic etiology. Neurology.

[59] Petkau TL, Leavitt BR (2014). Progranulin in neurodegenerative disease. Trends Neurosci.

[60] Kleinberger G (2013). Mechanisms of granulin deficiency: lessons from cellular and animal models. Mol Neurobiol.

[61] Whitwell JL (2009). Atrophy patterns in IVS10 + 16, IVS10 + 3, N279K, S305N, P301L, and V337M *MAPT* mutations. Neurology.

[62] Van Mossevelde S (2016). Clinical features of *TBK1* carriers compared with *C9orf72*, *GRN* and non-mutation carriers in a Belgian cohort. Brain.

[63] Van Deerlin VM (2007). Clinical, genetic, and pathologic characteristics of patients with frontotemporal dementia and progranulin mutations. Arch Neurol.

[64] Kelley BJ (2009). Prominent phenotypic variability associated with mutations in *Progranulin*. Neurobiol Aging.

[65] Pickering-Brown SM (2008). Frequency and clinical characteristics of progranulin mutation carriers in the Manchester frontotemporal lobar degeneration cohort: comparison with patients with *MAPT* and no known mutations. Brain.

[66] Beck J (2008). A distinct clinical, neuropsychological and radiological phenotype is associated with progranulin gene mutations in a large UK series. Brain.

[67] Rohrer JD (2010). Progranulin-associated primary progressive aphasia: a distinct phenotype?. Neuropsychologia.

[68] Carecchio M (2009). Progranulin plasma levels as potential biomarker for the identification of *GRN* deletion carriers: a case with atypical onset as clinical amnestic mild cognitive impairment converted to Alzheimer’s disease. J Neurol Sci.

[69] Carecchio M (2011). Cerebrospinal fluid biomarkers in Progranulin mutations carriers. J Alzheimers Dis.

[70] Pires C (2013). Phenotypic variability of familial and sporadic Progranulin p.Gln257Profs*27 mutation. J Alzheimers Dis.

[71] Rohrer JD (2009). Corticobasal syndrome associated with a novel 1048_1049insG progranulin mutation. J Neurol Neurosurg Psychiatry.

[72] Arosio B (2013). GRN Thr272fs clinical heterogeneity: a case with atypical late onset presenting with a dementia with Lewy bodies phenotype. J Alzheimers Dis.

[73] Pietroboni AM (2011). Phenotypic heterogeneity of the GRN Asp22fs mutation in a large Italian kindred. J Alzheimers Dis.

[74] Cerami C (2011). From genotype to phenotype: two cases of genetic frontotemporal lobar degeneration with premorbid bipolar disorder. J Alzheimers Dis.

[75] Rainero I (2011). Heterosexual pedophilia in a frontotemporal dementia patient with a mutation in the progranulin gene. Biol Psychiatry.

[76] Schymick JC (2007). Progranulin mutations and amyotrophic lateral sclerosis or amyotrophic lateral sclerosis-frontotemporal dementia phenotypes. J Neurol Neurosurg Psychiatry.

[77] Le Ber I (2008). Phenotype variability in progranulin mutation carriers: a clinical, neuropsychological, imaging and genetic study. Brain.

[78] Whitwell JL (2007). Voxel-based morphometry in frontotemporal lobar degeneration with ubiquitin-positive inclusions with and without progranulin mutations. Arch Neurol.

[79] Whitwell JL (2012). Neuroimaging signatures of frontotemporal dementia genetics: C9ORF72, tau, progranulin and sporadics. Brain.

[80] Whitwell JL, Josephs KA (2012). Neuroimaging in frontotemporal lobar degeneration – predicting molecular pathology. Nat Rev Neurol.

[81] Huey ED (2006). Characteristics of frontotemporal dementia patients with a Progranulin mutation. Ann Neurol.

[82] Boeve BF (2006). Frontotemporal dementia and parkinsonism associated with the IVS1 + 1G → A mutation in progranulin: a clinicopathologic study. Brain.

[83] Kovacs GG (2016). Aging-related tau astrogliopathy (ARTAG): harmonized evaluation strategy. Acta Neuropathol.

[84] Thal DR (2002). Phases of Aβ-deposition in the human brain and its relevance for the development of AD. Neurology.

[85] Braak H, Braak E (1991). Neuropathological stageing of Alzheimer-related changes. Acta Neuropathol.

[86] Mirra SS (1991). The Consortium to Establish a Registry for Alzheimer’s Disease (CERAD): Part II. Standardization of the neuropathologic assessment of Alzheimer’s disease. Neurology.

