# Supplementary material for: Extended FTLD pedigree segregating a Belgian GRN-null mutation: neuropathological heterogeneity in one family
Source: Alzheimers Res Ther. 2018 Jan 22;10:7. doi: 10.1186/s13195-017-0334-y (PMC6389176; doi:10.1186/s13195-017-0334-y)
Supplement: Supplementary file 1 — Materials and methods. (DOCX 26 kb) [file 13195_2017_334_MOESM1_ESM.docx]

**Additional File 1**

**Materials & Methods**

*Neuropathology*

In all our patients, brain was dissected, and specific regions were selected and paraffin embedded. Histochemical and advanced immunohistochemical analyses were performed. The following brain regions were sampled: Brodmann’s area 4 (gyrus precentralis), area 6 (gyrus frontalis superior), area 8 (frontal eye fields), area 11 (gyrus rectus), area 24 (gyrus cinguli), area 22 (gyrus temporalis superior), hippocampus, gyrus parahippocampalis, amygdala, area 7 (gyrus parietalis superior), area 17 (area striata), cerebellum, thalamus, neostriatum, pallidum, mesencefalon, pons, medulla oblongata.

White matter and vascular changes were examined in frontal cortex (Area 11 and adjacent white matter), hippocampus, temporal neocortex and adjacent white matter, anterior thalamus and internal capsule, area striata and cerebellum).

The arteries of the circle of Willis, when present, were carefully analyzed for atherosclerotic findings.

Histological analysis was performed with following stains: Haematoxylin-Eosin, Cresyl-Violet and Klüver-Barrera.

Immunohistochemistry was performed: Ubiquitin (Dako, Glostrup, Denmark), AT8 (against hyperphosphorylated tau (Innogenetics, Zwijnaarde, Belgium)), 4G8 (against β-amyloid (Signet, Dedham, Massachusetts)), α-synuclein (Sigma Aldrich, St Louis, Missouri), TDP-43 (Proteintech Group Inc, Chicago, Illinois), FUS (Proteintech Group Inc, Chicago, Illinois), P62 (BD Diagnostics, Erembodegem, Belgium), GFAP (antibody against glial fibrillary acidic protein (Dako, Glostrup, Denmark)). An additional staining to investigate small vessel changes included Masson’s trichrome.

*DNA extraction*

Confer [8] and [23] mutation analysis of all coding exons 1-12 and noncoding exon 0. 20 ng of genomic DNA was amplified by polymerase chain reaction (PCR) and amplification products were sequenced.

All patients were screened for C9orf72 repeat expansion, no expansion was detected. By use of an NGS gene panel based on high-level multiplex PCR amplification of targeted exons using the Multiplex Amplification of Specific Targets for Resequencing (MASTR) technology ([www.multiplicom.com](http://www.multiplicom.com)), followed by sequencing on a MiSeq platform (Illumina), all patients were screened for mutations in other mendelian genes associated with major neurodegenerative brain diseases phenotypes, i.e. Alzheimer’s disease, Frontotemporal dementia, Prion disease, ALS and Parkinson’s disease (APP, PSEN1, PSEN2, PRNP, C9orf72, MAPT, VCP, TARDBP, FUS, SOD1, LRRK2, PARK2 en SNCA). No mutations were revealed.

*Clinical history - Case reports*

All available clinical data were obtained from the neurologists in charge. Because the patients were treated by different neurologists in different centers, diagnostic approaches varied, so as the available clinical data. Of some of the patients, not all data were available (files lost, not digitalized…).

Age of disease onset is the age at which the subject presented the first symptoms of behavioral/personality changes, memory loss, problems in speech, motor changes or other neurological changes as noted by the patients themselves, their relatives or friends. This age of disease onset was mostly estimated by one single person, based on the information in the available clinical files. Disease duration was calculated from the (estimated) date of disease onset until time of death. Clinical symptoms and signs were reviewed, considered their chronological appearance. Final clinical diagnoses were in accordance with the current diagnostic criteria for the behavioral variant of FTD [1] and the diagnostic criteria for the variants of PPA [3].

*Neuropsychological assessment*

Global cognitive functioning was assessed using the Mini Mental State Examination. Neuropsychological assessment was not extensively performed in all subjects. Assessments were performed in different centers by different neuropsychologists and were mainly diagnosis driven. Depending on the main clinical characteristics, other assessments were done. Patient DR28.1 for example had a detailed, extensive neurolinguistic examination; patient DR2.3 for example had very limited neuropsychological testing due to verbal comprehension difficulties*.*

*Iconography*

During life time, all subjects had undergone imaging of the brain, structural and mostly functional as well. Most patients had had one or more structural Magnetic Resonance Imaging scans (MRI) of the brain. Of four patients, we had only knowledge of a structural CT scan (Computed Tomography). Concerning functional imaging, a SPECT (Single-photon emission computed tomography) of the brain was performed in seven patients, two patients had undergone a brain FDG-PET (fluoro-deoxy-glucose positron emission tomography), and in one patient (DR 31.1) functional imaging was not performed.
