# Supplementary material for: Extended FTLD pedigree segregating a Belgian GRN-null mutation: neuropathological heterogeneity in one family
Source: Alzheimers Res Ther. 2018 Jan 22;10:7. doi: 10.1186/s13195-017-0334-y (PMC6389176; doi:10.1186/s13195-017-0334-y)
Supplement: Supplementary file 2 — Results: neuropathology. (DOCX 22 kb) [file 13195_2017_334_MOESM2_ESM.docx]

**Additional File 2**

**Results: Neuropathology**

A summary of the macroscopical data can be found in Table A.

**Patient DR2.3**

Macroscopical analysis of the circle of Willis showed that the distal part of the basilar artery had one small plaque. Medial cerebral artery showed no changes.

Severe neuronal cortical loss was present, mainly in frontal and temporal cortices (area 6,9,22). In the temporal lobe, both neocortex (temporal superior gyrus – area 22), hippocampus and parahippocampal gyrus were affected. There was a neuronal loss of the substantia nigra, with status pigmentatus.

There was a mild thickening of the arteriolar walls, more explicit in basal ganglia, with moderate amount of perivascular hemosiderin deposit.

With immunohistochemistry, TDP-43 immunoreactive intracytoplasmic inclusions were found in the examined frontal cortices (area 6, 8, 9, 11), in the hippocampus and parahippocampal gyrus, in the parietal superior gyrus (area 7) and in the area striata (area 17). Sparse and occasional intranuclear inclusions were found in the above mentioned cortical areas. In the neostriatum, NCI (neuronal cytoplasmic inclusions) and NII (neuronal intranuclear inclusions) were present, however less extensive than in the cortical areas.

Findings were compatible with TDP proteinopathy type A.

We also found AT8 immunoreactive NFT (neurofibrillary tangles) in the parahippocampal gyrus and amygdala. 4G8 immunoreactive, beta-amyloid containing senile plaques were found in the parahippocampal gyrus, in the area striata (area 17), in the amygdala and in the gyrus temporalis superior (area 22). Findings were compatible with AD neuropathological diagnosis of A1B1C1.

There was a mild thickening of the arteriolar walls, more explicit in frontal white matter and basal ganglia, with moderate amount of perivascular hemosiderin deposits.

**Patient DR8.1**

Of the circle of Willis, only the medial cerebral artery was present, as well as the cerebellar arteries. There were no atherosclerotic changes.

A marked atrophy of the frontal cortices was observed in areas 8, 10, 30, 11, in the temporal neocortex (area 22), but also in the parietal cortex (area 7) and in the neostriatum. There were some ischemic changes in the thalamus. There was a neuronal loss and status pigmentatus of the substantia nigra. There was a mild to moderate neuronal loss in CA1 and subiculum.

There was a thickening of the walls of arterioles and small arteries, with perivascular hemosiderin deposit and perivascular space dilation. Vascular pathology was found in frontal and temporal regions as well in basal ganglia.

TDP-43 intracytoplasmic inclusions were found in the frontal cortices (prefrontal gyrus, cingulate gyrus, area 4, 8, 11). Sparse NII were found in the neurons of the second cortical layer in area 10 and 11.

NCI were also present in the superior temporal gyrus, and in the parietal area 7. No abnormalities were found in the area striata.

In the neostriatum NCI were also numerous. Lesions were compatible with TDP-43 proteinopathy type A.

A mild amount of AT8 immunoreactive NFT pathology was found in the parahippocampal gyrus and amygdala, compatible with Braak stage 1. There were no senile plaques. Findings were compatible with AD neuropathological changes A0B1C0.

**Patient DR25.1**

There was no circle of Willis present.

The neuronal loss was most pronounced in area 6 and 11, without abnormalities in the temporal or striatal areas. Hippocampal CA1 and subiculum showed mild neuronal loss. Parietal cortex could not be examined.

In the basal ganglia, there was severe atrophy and gliosis of the neostriatum and dorsomedial nuclei of the thalamus. Moderate neuronal loss in the substantia nigra and locus coeruleus. Small vessel changes with thickening of the arteriolar wall, perivascular hemosiderin deposits, perivascular demyelination were more explicit in frontal regions and in the hippocampal area.

TDP-43 immunoreactive NCI are present in the superior frontal gyrus, in area 4 and in area 11. Further sparse NCI were found in the parahippocampal gyrus.

In the basal ganglia, NCI were only present in the neostriatum.

NFT were found in CA1 and subiculum, there were no senile plaques. Findings were compatible with AD neuropathological changes A0B1C0

Lesions were compatible with TDP-43 proteinopathy type A.

**Patient DR25.5**

There were minimal atherosclerotic plaques in the basilar artery. No other arteries of the circle of Willis were present.

In the frontal cortex, there was an atrophy in areas 6,11,30 with normal findings in area 4 and 8. No neuronal loss in the temporal neocortex, parietal cortex or area striata.

Slight to moderate neuronal changes and gliosis in CA1 and subiculum of the hippocampus, and in the amygdala (basal nucleus). We noted a severe neuronal loss in the neostriatum (caudate nucleus). Normal findings in thalamus and other basal ganglia. Thickening of the arteriolar wall, dilatation of the perivascular space, hemosiderin deposits were found in the frontal and temporal lobes.

There was a moderate neuronal loss in the substantia nigra.

TDP-43 immunoreactive NCI, NII and neurites were found in areas 4, 6, 11, 30 of the frontal lobe. In temporal neocortex (area 22) NCI and NII were also present, however less numerous. There were sparse NCI in parietal cortex. No lesions in area striata.

The neostriatum was severely affected, with loads of NCI, NII and neurites.

Lesion load featured a diagnosis of TDP-43 proteinopathy type A.

Furthermore, moderate NFT were found in the CA3,2,1 and subiculum of the hippocampus and in the amygdala. No senile plaques, containing beta-amyloid could be detected. Findings were compatible with AD neuropathological changes A0B1C0.

**Patient DR28.1**

At the basal part of the brain, the right medial cerebral artery, the basilar artery, the right posterior communicating artery had no atherosclerotic changes.

The atrophy was most severe in the frontal areas 4, 6, 8, 11, 24, in the temporal superior gyrus (22). No atrophy or gliosis in the parietal or striatal areas. There was no cell loss in the hippocampus, CA1, subiculum, entorhinal cortex and lateral occipito-temporal gyrus. Mild atherosclerotic changes in frontal, temporal regions and basal ganglia.

There was a neuronal loss in the substantia nigra and in the dorsomedial formation of the thalamus. Neostriatum looks normal with histochemical analysis.

TDP-43 immunoreactive NCI, NII and neurofilaments were found in the involved frontal areas 6, 8, 11, 24, in the temporal area 22, in the parietal area 7. Sparse NCI in the lateral occipito-temporal gyrus. In the putamen, occasional NCI and NII were found, as in the medial formation of the thalamus.

Again, the TDP-43 pathology could be classified as a TDP43 proteinopathy type A.

NFTs were present in the entire hippocampus and parahippocampal gyrus, with the highest lesion load in the entorhinal cortex. Many NFT were found in the area striata, and sporadic NFT and neuritic threads in the substantia nigra and formation reticularis of the mesencephalon.

4G8 staining revealed senile plaques, of classic and diffuse type in the entire hippocampus and parahippocampal gyrus, as well as in the areas 4, 7, 8, 11, the cerebellar cortex and in the formation reticularis of the mesencephalon.

The combination of hyperphosphorylated tau pathology and beta-amyloid containing plaques suggested a neuropathological diagnosis A2B1C1.

**Patient DR205.1**

Analysis of blood vessels was not possible as no arteries of the circle of Willis were present after autopsy.

A moderate atrophy in the temporal and frontal cortices was found with slight neuronal loss in the CA1 region of the hippocampus and moderate neuronal loss in the substantia nigra. Parietal cortex could not be examined.

The neostriatum, thalamus and the other basal ganglia were normal. There was an occasional thickening of the arteriolar wall in the hippocampus and basal ganglia, with hemosiderin deposits and perivascular space dilatation.

AT8 staining demonstrated NFT in the lateral and medial occipito-temporal gyri, and sparse NFT, dystrophic neuritis and neuritic threads in the CA1, subiculum and entorhinal cortex.

Beta-amyloid plaques were present in the entire hippocampus, in the GOTL and GOTM, as well as in the insular cortex.

The presence of AT8 and 4G8 positive lesions favored a diagnosis of Alzheimer’s disease, neuropathological changes A2B1C1.

Alpha-synuclein staining shows extensive Lewy body pathology in the frontal and temporal cortices, in the hippocampus and in the parahippocampal gyrus. In the mesencephalon, Lewy bodies are present in the substantia nigra and in the formation reticularis. Locus caeruleus however could not be examined. As the classification of McKeith proposes, diagnosis of Lewy Body Disease, neocortical, could be made[50].

TDP43 pathology was extensively present in the GOTL, GOTM, in frontal and temporal cortices. In the hippocampus proper and in its dentate gyrus, subiculum, entorhinal cortex there were moderate NCI. In neostriatum NCI, NII and neurofilaments were numerous.

There was no TDP pathology in the mesencephalon, nor in the substantia nigra.

**Patient DR31.1**

Mild atherosclerotic changes in the posterior communicating artery, in the cerebral medial artery. Normal anterior cerebral artery. Normal aspect of cerebellar arteries. One small plaque in the basilar artery.

Histological analysis showed neuronal cell loss in the frontal cortices of areas 4, 6, 11, 24, in the temporal area 22 with a slight neuronal loss in the CA1 of the hippocampus, subiculum and entorhinal cortex.

A moderate cell loss was observed in the substantia nigra with status pigmentatus.

Areas 8 (frontal eye fields), 7 (somatosensory association cortex in parietal lobe), area striata, thalamus and neostriatum revealed no abnormalities. Mild cerebrovascular pathology with thickening of the vessel media, with perivascular hemosiderin deposits and perivascular space dilatation.

TDP43- staining shows numerous of NCI, NII and NF in area 8 and 11 and a moderate TDP pathology in areas 4, 6 and 24. No alterations in the temporal area 22. Sparse NCI presence in parietal lobe and area striata and amygdala.

In the basal ganglia, numerous TDP-43 NCI and NII were present in the neostriatum. Thalamus (medial formation) was only mildly involved.

AT8 showed rare NFT in CA1, subiculum, entorhinal cortex and amygdala. No beta-amyloid pathology was observed. Findings were compatible with AD neuropathological changes A0B1C0.

**Patient DR1207.1**

Circle of Willis was not present.

Severe cortical loss was found in areas 24, 6 and 8 of the frontal lobe, more than in areas 9 and 10, in the superior temporal gyrus and for a lesser extent in the mesotemporal cortex. There was mild atrophy of the parietal cortex. In the basal ganglia, we saw a severe atrophy of the caudatum, less explicit atrophy of the putamen, and mild atrophy of the medial formation of the thalamus. There was a mild thickening of the arteriolar walls, with moderate amount of perivascular hemosiderin deposit, myelin loss and dilatation of the perivascular space. Vascular pathology was more present in frontal lobe.

TDP pathology was present in neostriatum, frontal and temporal cortices. Minimal pathology was found in parietal cortex and in striatal area. TDP pathology included mainly NCI and DN in the second cortical layers, NII ware sporadic.

There was minimal hyperphosphorylated tau pathology in the hippocampal and parahippocampal structures. Beta-amyloid pathology was absent. Findings were compatible with AD neuropathological changes A0B1C0.

**Patient DR1213.1**

Inspection of the arteries of the circle of Willis showed no atherosclerotic changes.

There was a severe cortical atrophy, with cortical thickness less than 1mm. Areas that most atrophic were cingulate gyrus (area 24), gyrus rectus (area 11), areas that were less atrophic were area 6, 8, 10. Parietal cortex was also atrophic, as well as the superior temporal gyrus and the parahippocampal gyrus. In the basal ganglia, there was a severe neuronal loss and atrophy of the head of the caudate nucleus, more than the putamen. Again, medial formation of the thalamus was also atrophic. No abnormalities in the Betz neurons, nor the neurons in the hypoglossal nucleus. Thickening of the arteriolar wall was more pronounced in basal ganglia with hemosiderin deposit and perivascular space dilatation.

TDP stain showed loads of NCI and dystrophic neurites, mainly in the superficial cortical layers of the affected cortical areas. Occasional NII were found in cortex, caudatum and transentorhinal cortex.

NFT pathology was found in the amygdala and parahippocampal gyrus (Braak stage 1), whereas there was no beta-amyloid pathology. These findings are compatible with AD neuropathological diagnosis A0B1C0.

**Table A. Macroscopical findings**

| Atrophy in | DR2.3 | DR8.1 | DR25.1 | DR25.5 | DR28.1 | DR205.1 | DR31.1 | DR1207.1 | DR1213.1 |
| --- | --- | --- | --- | --- | --- | --- | --- | --- | --- |
| Frontal lobe | +++ | +++ | +++ | +++ | +++ | NA | + | +++ | +++ |
| Temporal lobe | +++ | + | + | +++ | + | NA | + | ++ | + |
| Parietal lobe | + | - | ++ | - | +++ | NA | - | - | - |
| Nucleus caudatus | + | + | NA | + | + | NA | + | +++ | +++ |
| Whole cortical atrophy | + | - | - | - | - | NA | - | - | - |
|  |  |  |  |  |  |  |  |  |  |
| Weight of right hemisphere | 414 g | 519 g | 449 g | 472 g | 583 g | NA | NA | 522 g | 577 g |

*Legend: NA (not available), + (mild atrophy), ++ (moderate atrophy); +++ (severe atrophy)*
