# Supplementary material for: Extended FTLD pedigree segregating a Belgian GRN-null mutation: neuropathological heterogeneity in one family
Source: Alzheimers Res Ther. 2018 Jan 22;10:7. doi: 10.1186/s13195-017-0334-y (PMC6389176; doi:10.1186/s13195-017-0334-y)
Supplement: Supplementary file 3 — Results: clinical and neuropsychological assessment. (DOCX 24 kb) [file 13195_2017_334_MOESM3_ESM.docx]

**Additional File 3.**

**Results: Clinical and neuropsychological assessment**

The major clinical and neuropsychological data are summarized in Table B.

**Table B. Clinical and neuropsychological assessment**

| Patients | DR2.3 | DR8.1 | DR25.1 | DR25.5 | DR28.1 | DR205.1 | DR31.1 | DR1207.1 | DR1213.1 |
| --- | --- | --- | --- | --- | --- | --- | --- | --- | --- |
| Behavioral disinhibition |  | 1 |  |  | 2 | 1 |  |  |  |
| Apathy or inertia | 2 | 1 | 1 | 1 | 2 | 2 |  | 2 | 1 |
| Hyperorality |  | 1 | 1 |  | 2 |  |  |  |  |
| Perseverative, stereotyped behavior | 2 | 1 | 1 |  |  |  |  |  |  |
| Psychotic features |  | 1 |  |  |  |  |  |  |  |
| Memory loss | 1 | 1 | 2 | 1 |  | 2 |  |  | 2 |
| Economy of speech | 1 | 1 | 1 | 1 | 1 | 2 | 1 | 2 | 1 |
| Oral apraxia or agrammatism | 1 |  |  | 1 | 1 | 2 | 1 | 1 |  |
| Comprehension deficits | 2 |  | 2 | 1 |  |  |  |  |  |
| Naming deficits | 1 | 1 | 2 |  | 1 |  |  |  |  |
| Akinesia |  | 1 | 1 |  |  | 2 |  |  |  |
| Tremor |  |  |  | 1 | 2 | 1 |  | 2 |  |
| LMN signs |  |  |  |  |  |  |  |  |  |
| UMN signs | 2 |  |  | 1 |  |  |  | 2 |  |
| Primitive reflexes | 2 |  | 1 |  |  | 2 |  | 1 | 1 |
| Limb apraxia |  | 1 | 1 |  |  |  |  | 2 |  |
| Oculomotor palsy |  |  |  |  |  |  |  |  |  |
| Cog Wheel rigidity or other parkinsonian symptoms |  |  |  |  | 2 | 1 |  | 2 |  |

*Legend: 1 (symptom present in the initial phase of the disease), 2 (symptom present in a later stage of the disease)*

**Patient DR 2.3**

Since the age of 63.5 years old, this patient had progressive complaints of forgetfulness with naming and word retrieval deficits. Despite the complaints of forgetfulness, the patient performed still well on the MMSE at the age of 64, with a score of 27/30. After a TIA with aphasia at the age of 64, the problems in speech evolved quite quickly to a speech characterized by stuttering, grammatical difficulties and word retrieval deficits at the age of 65. At that age, she also showed serious disturbances in calculation, concentration and abstraction, and moderately disturbed short memory reproduction. Clinical neurological examination at the age of 65 was still normal. At the age of 67 (disease duration (DD) 4 years), speech was non-fluent with evidence of pronounced comprehension deficits which limited the possibilities for neuropsychological testing. By that time, she scored 9/30 on the MMSE. Clinical examination at the age of 67, showed brisk reflexes with doubtful plantar reflex in extension on the right and frontal release signs such as a snout reflex and a bilateral grasp reflex. During the disease, the patient showed changes in personality and character with rigidity in thinking and apathy, and progressively she became more dependent. Since the initial clinical picture of this patient was dominated by problems in speech characterized by non-fluency and grammatical difficulties, we conclude to a final diagnosis of the agrammatic variant of PPA.

**Patient DR 8.1**

This patient started to complain of word retrieval difficulties and short-memory problems at the age of 62, after a TIA with central paresis of the VIIth cranial nerve. Performance on the MMSE was still well with a score of 28/30. Soon more problems were reported and one year after disease onset (age of 63) there were features of frontal dysfunction such as sleepiness, confabulation, loss of initiative and activity, hyperorality with bulimia, loss of decorum, perseveration, attention deficits and akinesia. Also, features of temporal dysfunction (auditive hallucinations, progressive memory loss) and parietal dysfunction (disorientation, apraxia) were present. Speech was characterized by echolalia and mutism, and he obtained an MMSE score of 23/30 (DD 1 year). Since behavioral problems were the initial and main problem, the final clinical diagnosis in this patient was behavioral FTD.

**Patient DR 25.1**

Problems started at the age of 69 with progressive taciturnity and frontal behavioral problems such as loss of initiative and bulimia. Urinary incontinence and parietal features of clothing apraxia and environmental agnosia were reported as well. Three years after disease onset, there were clear signs of frontal dysfunction such as a total loss of initiative, absence of spontaneous speech with the existence of pronounced echolalia, perseverations and confabulations. There was a tendency to akinesia and mutism, although there were repetitive movements of the hands. Primitive frontal reflexes such as glabella reflex, snout reflex and grasp reflex, were positive during clinical examination three years after disease onset. Memory loss was not reported as a primary symptom but three years after disease onset, there was evidence of memory loss with low scores for memory in the hierarchic dementia scale and an MMSE score of 16/30. In addition, neuropsychological testing at that time (DD 3 years) showed evidence for comprehension deficits, naming deficits (mostly anomic), but also for dyslexia, dysgraphia, acalculia, apraxia (ideomotor and constructional) and disorientation, indicating parietal involvement. Some answers during neuropsychological testing were inappropriate. Since behavioral problems were the main primary problem and significant difficulties in speech appeared only later in the disease progress, the final clinical diagnosis in this patient was behavioral FTD.

**Patient DR 25.5**

Initial complaints started at the age of 70 and consisted of: progressive memory deficits, signs of frontal dysfunction such as global apathy, sleepiness and progressive difficulties in speech which evolved to barely spontaneous speech one year after disease onset. Urinary incontinence, increased emotionality, comprehension deficits and gait problems were reported as well. Clinical neurological examination showed hyperreflexia on the right, dragging of the right lower limb, and tremor and rigidity in the right upper limb. One year before onset of his dementia, this patient suffered from a stroke in the left ACM region. This stroke explains the right hyperreflexia and dragging of the right leg. Due to the stroke, there was already a preexistence of phatic problems, which worsened during his dementia. The final clinical diagnose of this patient was behavioral FTD.

**Patient DR 28.1**

The first symptoms started at the age of 56 and consisted of isolated progressive speech difficulties without evidence for other cognitive problems. An extensive neurolinguistic assessment two and a half years after disease onset, concluded to a diagnosis of primary progressive aphasia (agrammatic variant) with normal general cognitive functioning (MMSE 28/30). The speech difficulties were characterized by non-fluent speech with multiple phonematic paraphasias, palilalia, impaired repetition of sounds, words and sentences with relatively spared comprehension. There were features of parietal dysfunction such as agraphia, apraxia in writing, acalculia and right/left confusion without finger agnosia. Progressively, speech declined further and cognitive and behavioral problems appeared. Decreased activity, demotivation, decreased concentration, decreased sense of reality and visual naming disorders were reported three years after disease onset. One year later, he was on the one hand adynamic with loss of initiative and decreased expression of emotions, but on the other hand, he could act impulsive and was easily angry or agitated. By that time, he was mute and showed hyperorality with weird eating habits. He also had features of temporal dysfunction such as problems in auditory and reading comprehension. His score on MMSE was declined to 19/30 three years after disease onset and to a score of 12/30 one year later. In the evolution of the disease, the patient developed parkinsonian features such as resting tremor, stooped posture, sialorrhoea, shuffling gate. Bulbar problems appeared and placement of a PEG tube was needed four and a half years after disease onset. The final clinical diagnosis was PPA-agrammatic.

**Patient DR 205.1**

Since the age of 55, this patient suffered of a resting tremor in the right hand and to a lesser extent in the right foot. Other parkinsonian symptoms during clinical neurological examination at that time were cogwheel rigidity, moderate diffuse bradykinesia and reduced arm swing. Although clinical neuropsychological assessment at that time didn’t show any evident problems, the patient complained of some difficulties in speech, concentration, memory and writing, and of increased tiredness and reduced emotional inhibition with crying without a clear reason. The parkinsonian symptoms improved initially well with levodopa treatment and one year after onset the patient was diagnosed with Parkinson’s disease. During the following years, there was further reporting of frontal dysfunction with decreased dynamics and spontaneous speech, increased tiredness and depressed mood. Three years after onset of his Parkinson’s disease, frontal dysfunction was more evident with clear presence of apathy, loss of spontaneous speech, neurologic stuttering and echolalia, urinary incontinence, changes in sexual behavior and impaired emotional control. Neuropsychological and neurolinguistic assessment at that time showed severe problem solving disorders, severe language disorders and severe problems in encoding short-term memory. There was also evidence of parietal involvement since disorientation, disorders in gnosis and ideational and constructional apraxia were present as well. There was a fast cognitive decline with an MMSE score of 11/30 at the age of 58 years and 9 months, which evolved to a score of 3/30 two months later. By that time, he was relatively mute and showed primitive frontal release signs (bilateral pathologic glabella reflex, grasp reflex, palmomental reflex and snout reflex). The parkinsonian symptoms were evolved to global rigidity with bilateral cogwheel rigidity, hypokinesia, hypophonia and disturbed shuffling gate with antiversion and retropulsion. By the age of 60, the patient was complete mute, wheelchair-bound and totally dependent. The final clinical diagnose of this patient was Parkinson’s disease with very severe frontal dysfunction.

**Patient DR 31.1**

The complaints started insidious at the age of 65, with subjective progressive difficulties in word retrieval. Neurolinguistic and neuropsychological assessment 1 year after disease onset showed a normal cognitive function with absence of memory loss, apraxia or agnosia. Speech was disturbed with non-fluent, reluctant articulation and self-corrected phonematic paraphasias. Testing of word retrieval, comprehension, reading and writing was normal. One year later (DD 2 years) neuropsychological assessment of orientation, memory, praxis, gnosis, concentration was still normal. Meanwhile, testing of frontal function gave clearly abnormal results in frontal problem solving and frontal attention. Clinical neurological examination three years and a half after disease onset was still normal, there were no pathological primitive reflexes or parkinsonian symptoms. The final diagnosis in this patient was the agrammatic variant of primary progressive aphasia.

**Patient DR1207.1**

At the age of 62, this patient developed progressive difficulties in speech. Around that time, the patient also caused a car accident. No behavioral or memory symptoms were reported. A neuropsychological examination performed around that time, was characterized by a systematic discrepancy to the detriment of verbal mediated cognitive functions. There was a dysexecutive syndrome with pathologically decreased attention and problem solving. Apraxia in speech was noticed and the diagnosis of the agrammatic variant of primary progressive aphasia was made. One year and a half after disease onset, gait deficits started. Clinical neurological examination two years after disease onset demonstrated not only the presence of frontal disinhibition reflexes but also mild cog wheel rigidity most pronounced on the right, a discrete resting tremor in the right upper limb and an odd gait pattern with dragging of the right leg, which was interpreted as a symptom of apraxia. Three months later, a Babinski sign was observed on the left. Since DAT (dopamine transporter) imaging was compatible with a real Parkinson syndrome, levodopa was initiated. However, no amelioration of the extrapyramidal symptoms was remarked. CSF (cerebrospinal fluid) biomarkers were not compatible with Alzheimer’s disease. Motor and cognitive functions deteriorated progressively and around four years after disease onset, the patient was almost mute and unable to march independently.

**Patient DR1213.1**

Initial complaints of low energy, emotional bluntness and lack of initiative, started at the age of 58. Furthermore, the patient experienced reduced memory skills and restlessness was mentioned. Eating habits changed with an increased preference for sweet foods. Neuroimaging and CSF biomarkers were compatible with FTLD, leading to a diagnosis of behavioral variant FTD. Apathy increased progressively and cognitive functions such as memory, problem solving and orientation in time decreased. The patient died at the age of 60 after euthanasia.
