# Supplementary material for: Extended FTLD pedigree segregating a Belgian GRN-null mutation: neuropathological heterogeneity in one family
Source: Alzheimers Res Ther. 2018 Jan 22;10:7. doi: 10.1186/s13195-017-0334-y (PMC6389176; doi:10.1186/s13195-017-0334-y)
Supplement: Supplementary file 4 — Results: imaging data. (DOCX 16 kb) [file 13195_2017_334_MOESM4_ESM.docx]

**Additional File 4.**

**Results: Imaging data (Table C)**

**Patient DR2.3**

A CT scan of the brain, half a year after disease onset (63 years) showed cortical atrophy. MRI of the brain two years after disease onset didn’t show any clear abnormalities except some aspecific, probably ischemic white matter lesions periventricular bifrontal, more pronounced in the left frontal region. Later structural scans (DD 4-5 years) of the brain showed enlarged ventricles suggestive for normal pressure hydrocephalus (NPH), but presumably the enlargement was based on atrophy since NPH was not confirmed by intracranial pressure measurement. A SPECT of the brain three and a half years after disease onset showed pronounced hypoperfusion in the right frontal region and less pronounced hypoperfusion in the left frontal and bilateral temporal regions.

**Patient DR8.1**

At the age of 63, four months after disease onset, a CT scan of the brain showed cortical and subcortical atrophy. An MRI of the brain one year later, showed moderate frontal atrophy, periventricular vascular white matter lesions (left>right) with dilatation of the lateral ventricles and the third ventricle suggestive for normal pressure hydrocephalus. The latter was confirmed on MRI with flow measuring. A SPECT of the brain one year after disease onset, showed bifrontal hypoperfusion, more pronounced on the left side.

**Patient DR25.1**

A CT scan of the brain three years after disease onset, showed atrophy, most pronounced frontal periventricular and to a lesser extent temporal periventricular, with extensive periventricular white matter lesions especially frontal*.* Functional imaging with SPECT (DD 3 years) showed severe hypoperfusion bifrontal, moderate hypoperfusion biparietotemporal and hypoperfusion of the basal ganglia, especially of the right thalamus. There was no clear asymmetry on imaging, but an EEG (DD 3 years) showed increased irritative slow wave activity in the temporal regions that was more pronounced on the left.

**Patient DR25.5**

A structural MRI of the brain one year after disease onset showed severe cortical and subcortical atrophy particularly frontal and in the left hemisphere, and chronical vascular white matter lesions especially in the frontal regions, also predominantly on the left. On a whole body PET performed one year after disease onset there was important hypometabolism in several cerebral regions (bilateral frontal, parietotemporal and in the region of the basal ganglia on the left) and in the right cerebellar hemisphere. The hypometabolism in the left cerebral hemisphere is at least partially a result of the stroke.

**Patient DR28.1**

A structural MRI of the brain nine months after disease onset showed limited global atrophy. Functional imaging with SPECT two and a half year after disease onset showed a relative frontoparietal hypoperfusion more pronounced on the left, with extension to the left temporal region.

**Patient DR205.1**

Structural imaging with CT, three years after disease onset, showed severe atrophy, especially bilateral frontal and parietal. SPECT of the brain (DD 3 years) showed severe hypoperfusion bifrontal, more pronounced on the left, and, to a lesser extent, hypoperfusion biparietal, extending to bitemporal.

**Patient DR31.1**

This patient had only structural imaging performed. A CT of the brain, three and a half years after disease onset, showed mild global cerebral cortical atrophy.

**Patient DR1207.1**

On a SPECT (DD 1 year) of the brain, hypoperfusion was present in the frontal, and to a lesser extent, in the temporal lobes, more pronounced on the left. Furthermore, hypoperfusion was present in the basal ganglia and thalamus of the left hemisphere. On a MRI of the brain (DD 2 years), periventricular white matter lesions were present, especially in the left hemisphere, and cortical atrophy was most pronounced in the left frontal and temporal regions. On a DAT-scan (DD 1 year), there was hypocaptation in the left putamen, compatible with a real Parkinson syndrome.

**Patient DR1213.1**

A structural MRI one year after disease onset, showed predominant frontal and to a lesser extent parietal brain atrophy. At the same time, SPECT imaging showed hypoperfusion in the right frontal lobe. On FDG-PET imaging two months later, severe decreased metabolism was present in both frontal cortices, more pronounced on the right side, and to a lesser extent in the right temporal cortex.

**Table C. Structural and functional imaging**

| Patient | CT/MRI | SPECT/FDG-PET | Asymmetry | Parietal involvement |
| --- | --- | --- | --- | --- |
| DR2.3 | CT (DD0.5) cortical atrophy  MRI (DD2): aspecific WML periventricular bifrontal (L>R) | SPECT (DD3.5): frontal; bilateral temporal hypoperfusion (R>L) | Y (R>L) |  |
| DR8.1 | CT (DD0): cortico subcortical atrophy; MRI (DD1): frontal atrophy with periventricular WML (L>R) | SPECT (DD1): frontal hypoperfusion (L>R) | Y (L>R) |  |
| DR25.1 | CT (DD3): frontotemporal atrophy (frontal > temporal), frontal periventricular WML | SPECT (DD3): Hypoperfusion bilateral frontal > bilateral parietotemporal > basal ganglia (thalamus R). | N | Y |
| DR25.5 | MRI (DD1): severe cortico subcortical atrophy (L>R), WML bifrontal (L>R) | FDG-PET (DD1): hypometabolism bifrontal, parietotemporal, basal ganglia on the left and right cerebellum | Y (L>R) | Y |
| DR28.1 | MRI (DD0.5): limited global atrophy | SPECT (DD2.5): hypoperfusion frontoparietal L> R extending to left temporal region) | Y (L>R | Y |
| DR205.1 | CT (DD3): frontal and parietal atrophy | SPECT (DD3): severe hypoperfusion bilateral frontal (L>R), bilateral parietal, evolving to temporal | Y (L>R) | Y |
| DR31.1 | CT (DD3): mild cortical atrophy | ND | NA | NA |
| DR1207.1 | MRI (DD2): frontotemporal cortical atrophy (L>R), periventricular WML (L>R) | SPECT (DD1): frontal > temporal hypoperfusion (L>R), hypoperfusion of left basal ganglia and left thalamus | Y (L>R) |  |
| DR1213.1 | MRI (DD1): frontal > parietal atrophy | SPECT (DD1): frontal R hypoperfusion; FDG-PET (DD1.2): severe hypometabolism bifrontal (R > L), lesser hypometabolism R temporal | Y (R>L) | Y |

*Legend: DD (disease duration in years); NA (not applicable); L (left); R (right), WML (white matter lesions)*
